# The Regulation of AMPA Receptor Endocytosis by Dynamic Protein-Protein Interactions

**DOI:** 10.3389/fncel.2018.00362

**Published:** 2018-10-11

**Authors:** Jonathan G. Hanley

**Affiliations:** Centre for Synaptic Plasticity and School of Biochemistry, University of Bristol, Bristol, United Kingdom

**Keywords:** synaptic plasticity, LTD (long term depression), clathrin, AP2 clathrin adaptor complex, PICK1, protein interacting with C-kinase 1

## Abstract

The precise regulation of AMPA receptor (AMPAR) trafficking in neurons is crucial for excitatory neurotransmission, synaptic plasticity and the consequent formation and modification of neural circuits during brain development and learning. Clathrin-mediated endocytosis (CME) is an essential trafficking event for the activity-dependent removal of AMPARs from the neuronal plasma membrane, resulting in a reduction in synaptic strength known as long-term depression (LTD). The regulated AMPAR endocytosis that underlies LTD is caused by specific modes of synaptic activity, most notably stimulation of NMDA receptors (NMDARs) and metabotropic glutamate receptors (mGluRs). Numerous proteins associate with AMPAR subunits, directly or indirectly, to control their trafficking, and therefore the regulation of these protein-protein interactions in response to NMDAR or mGluR signaling is a critical feature of synaptic plasticity. This article reviews the protein-protein interactions that are dynamically regulated during synaptic plasticity to modulate AMPAR endocytosis, focussing on AMPAR binding proteins and proteins that bind the core endocytic machinery. In addition, the mechanisms for the regulation of protein-protein interactions are considered, as well as the functional consequences of these dynamic interactions on AMPAR endocytosis.

## Introduction

Since AMPA receptors (AMPARs) mediate the majority of fast synaptic excitation in the central nervous system, their regulation at the synapse is of fundamental importance to brain function. The formation of neuronal circuits during brain development and their subsequent modification during learning, forgetting and other aspects of memory processes require plasticity at excitatory synapses in the brain, manifested by changes in synaptic strength (Chater and Goda, 2014; Henley and Wilkinson, 2016). Long-term potentiation (LTP; an increase in synaptic strength) and long-term depression (LTD; a decrease in synaptic strength) are synapse-specific (Hebbian) forms of plasticity that have been the subject of intense research for many years and are now considered to be the major mechanisms that underlie such changes (Huganir and Nicoll, 2013). In addition, homeostatic plasticity, also known as synaptic scaling, involves a cell-wide adjustment of synaptic strength to maintain a stable output of a particular neuron during changes in neuronal circuit activity (Fernandes and Carvalho, 2016).

A major component of these forms of synaptic plasticity is the trafficking of AMPARs to or from synapses to increase or decrease the number of AMPARs localized at synapses, and hence modulate the strength of synaptic transmission. The subject of this review article is AMPAR endocytosis, the consequence of which is the removal of receptors from the neuronal surface and hence from the synapse, leading to a decrease in synaptic strength (LTD). This process is essential for specific types of learning and memory systems (Griffiths et al., 2008; Connor and Wang, 2016; Migues et al., 2016). The precise regulation of AMPAR trafficking and hence of synaptic transmission is critical for the balance between maintaining memories/learned behaviors and modifying memories or storing new ones. In addition, a number of neurological disorders involves aberrant recruitment of AMPAR endocytosis mechanisms. This can cause pathological levels of synaptic depression or the internalization of specific AMPAR subtypes from the synapse as part of a process that results in the synaptic expression of Ca^2+^-permeable AMPARs, which contribute to neuronal death (Hsieh et al., 2006; Liu et al., 2006; Dixon et al., 2009).

AMPARs are complexes comprising the core pore-forming subunits GluA1–4, as well as an increasing number of auxiliary subunits that play critical roles in regulating various aspects of AMPAR function (Henley and Wilkinson, 2016; Greger et al., 2017; Jacobi and von Engelhardt, 2018). Core and auxiliary subunits are integral membrane proteins and are subject to the basic cell biological trafficking processes of endocytosis, endosomal sorting, recycling and exocytosis that apply to the majority of transmembrane proteins in most mammalian cell types. In this review article, I will discuss the current state of knowledge about specific mechanisms of AMPAR endocytosis, focussing on dynamic protein-protein interactions modulated by signaling pathways downstream of synaptic stimuli that induce long-term changes in synaptic transmission. While much is known about how dynamic protein-protein interactions are orchestrated and regulated in the generalized endocytic process (McMahon and Boucrot, 2011; Daumke et al., 2014) surprisingly few protein interactions have been identified that are regulated by plasticity stimuli to control AMPAR endocytosis, despite the intensity of research into synaptic plasticity mechanisms in the past two decades.

AMPARs are thought to be rarely static, but instead are continually cycling between the synapse and the endosomal system (Luscher et al., 1999; Ehlers, 2000; Lee et al., 2004). In a process thought to be largely driven by the GluA2 subunit and its associated proteins, AMPARs diffuse laterally from the synapse and are endocytosed at plasma membrane sites adjacent to the post-synaptic density (PSD), proposed to be specialized endocytic zones (EZs; Lu et al., 2007; Opazo and Choquet, 2011). Following sorting in the early endosome, AMPARs are either targeted for degradation in lysosomes or recycled to the plasma membrane, with reinsertion taking place away from the PSD and lateral diffusion in the plane of the membrane resulting in the reincorporation of AMPARs at the synapse (Opazo and Choquet, 2011; van der Sluijs and Hoogenraad, 2011). This review article will not discuss the details of AMPAR endosomal sorting, which is also a critical determinant of synaptic strength and is itself subject to regulation as an important aspect of synaptic plasticity. Moreover, it is important to note that experimental quantification of AMPAR “internalization,” for example in surface biotinylation or antibody-feeding assays, does not measure endocytosis *per se*, but is confounded by the amount of receptors that are retained in endosomal compartments or recycled to the plasma membrane. For example, dissociating a protein-protein interaction that blocks the NMDA-induced loss of surface AMPARs could be explained by an increase in recycling back to the plasma membrane as well as by a blockade of endocytosis. This review article will focus on mechanisms that have been specifically implicated in regulating AMPAR endocytosis.

LTD is typically induced by stimulation of either NMDA receptors (NMDARs) or metabotropic glutamate receptors (mGluRs), resulting in the activation of numerous Ca^2+^-dependent signaling cascades (Collingridge et al., 2010). The vast majority of dynamic protein-protein interactions in the regulation of AMPAR endocytosis have been defined in the context of NMDAR-dependent LTD in hippocampal neurons. While NMDAR- and mGluR-dependent forms of LTD are mechanistically similar, they differ in upstream signaling pathways, and consequently in some of the protein-protein interactions involved. However, there is insufficient evidence to completely define the distinct processes of mGluR- and NMDAR-dependent AMPAR endocytosis from the point of view of dynamic protein-protein interactions. While LTD is an important form of synaptic plasticity in the cerebellum as well as in forebrain neurons, hippocampal neurons have been more extensively investigated because at least until very recently, mechanistic cell biology studies have been better suited to cultured neurons than brain slice or *in vivo* preparations, and cerebellar Purkinje neurons are technically difficult to culture compared to hippocampal neurons. However, a number of protein-protein interactions that have been implicated in cerebellar LTD have been more fully defined as playing a role in AMPAR endocytosis in hippocampal neurons, and therefore it could be inferred that they are similarly involved in the cerebellum.

The mechanisms that underlie constitutive AMPAR endocytosis have much in common with activity-dependent endocytosis during LTD from the point of view of the protein-protein interactions involved. In fact, a number of protein-protein interactions that are either required for or restrict constitutive AMPAR endocytosis are up- or down-regulated in order to increase trafficking for LTD, and it is this concept that forms the core of this review. Nevertheless, while the majority of activity-dependent AMPAR endocytosis is thought to be clathrin and dynamin-dependent, some forms of constitutive AMPAR trafficking may proceed via clathrin and dynamin-independent mechanisms (Glebov et al., 2015), the details of which are beyond the scope of this review.

AMPAR subunits interact with a large (and still increasing) number of identified proteins, which facilitate and direct their trafficking between the synapse and the endosomal system. These accessory proteins in turn interact with other binding partners that integrate them into fundamental cell biological systems such as the actin cytoskeleton or the core endocytic machinery. The highly complex process of recruiting AMPARs to sites of endocytosis, and facilitating their internalization requires the up- or down-regulation of several protein-protein interactions in response to intracellular signaling initiated by NMDAR or mGluR stimulation. While the primary focus of this review is the protein-protein interactions involved in endocytosis *per se*, other interactions that precede endocytosis must be regulated for endocytosis to proceed, so are also discussed here.

## Dissociation From PSD Scaffolds

**Figure 1:**
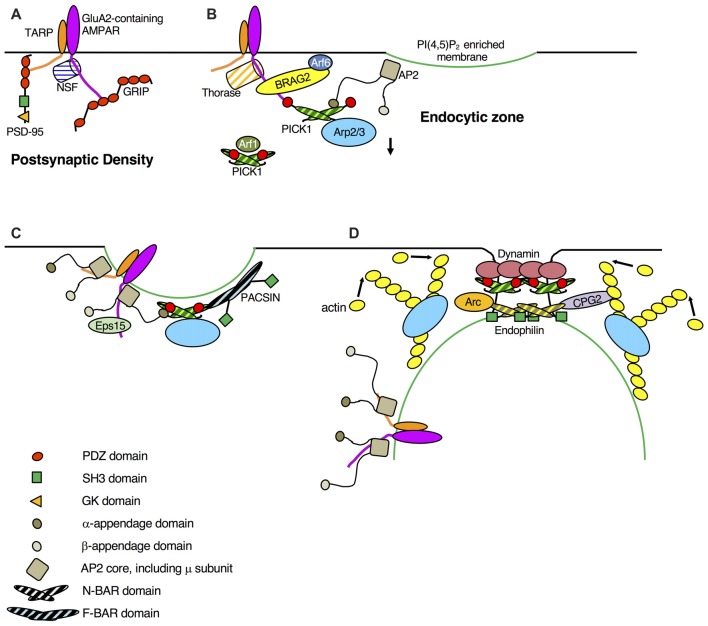
Schematic showing dynamic protein-protein interactions in AMPA receptor (AMPAR) endocytosis. **(A)** GluA2-containing AMPARs at the synapse are bound to post-synaptic density-95 (PSD-95) via transmembrane AMPAR regulatory proteins (TARPs) and to GRIP via GluA2. NSF activity prevents protein interacting with C-Kinase 1 (PICK1) binding to GluA2. **(B)** As a result of long-term depression (LTD) induction (NMDA receptor (NMDAR) or metabotropic glutamate receptor (mGluR) stimulation), TARP dephosphorylation disrupts TARP-PSD-95, GluA2 S880 phosphorylation and Thorase activity disrupt GluA2-GRIP. Ca^2+^ directly enhances GluA2-PICK1 and disrupts GluA2-NSF, deactivation of Arf1 promotes PICK1-Arp2/3 (inactive). GluA2 Y876 dephosphorylation enhances GluA2-Brefeldin-Resistant Arf-G2 (BRAG2), which in turn activates Arf6, causing a local increase in PI(4,5)P_2_ concentration, and consequent clustering of AP2. Calcineurin activity enhances AP2(α)-PICK1 to initiate AMPAR recruitment to clathrin-coated pits (CCPs). **(C)** TARP dephosphorylation enhances TARP-AP2(μ), and an unknown mechanism, possibly involving Hippocalcin, enhances GluA2-AP2(μ), both of which further promote AMPAR clustering at CCPs. AP2(α)-PICK1 interaction disrupts GluA2-PICK1. PACSIN phosphorylation enhances PICK1-PACSIN, which may stabilize curvature of the nascent CCP. Eps15 binds GluA1 in a ubiquitin-dependent manner. **(D)** As the complex geometry of the CCP develops, Bin-Amphiphysin-RVS (BAR) domain proteins stabilize the tight curvature of the CCP neck and recruit dynamin and other proteins to this structure. Calcineurin activity enhances PICK1-dynamin, activity-dependent increases in Arc and CPG2 expression enhance Endophilin-Arc and Endophilin-CPG2. CPG2 phosphorylation enhances CPG2-actin. Competition with Arp2/3 activators (e.g., N-WASP) disrupts PICK1-Arp2/3. Note that this schematic is limited to protein-protein interactions shown to be dynamically regulated in response to plasticity-inducing stimuli.

The PSD contains a multitude of scaffolding and signaling proteins involved in maintaining and regulating synaptic transmission (Feng and Zhang, 2009). PSD-95 functions as a “slot protein,” defining a place for an AMPAR at the synapse, and it is thought that the number of PSD-95 molecules localized to the PSD plays an important role in maintaining the number of AMPARs at that synapse (Opazo et al., 2012; Won et al., 2017). AMPARs interact with the PDZ domains of PSD-95 via the C-terminal tail of transmembrane AMPAR regulatory proteins (TARPs), the most-studied family of AMPAR auxiliary subunit, of which Stargazin is the prototypical member (Chen et al., 2000; Figure 1A). The TARP—PSD-95 interaction reduces the lateral mobility of AMPARs at the synapse, and disrupting this interaction allows AMPARs to diffuse away from the synapse, still bound to TARPs (Bats et al., 2007). The TARP—PSD-95 interaction is dynamic and subject to regulation by phosphorylation of a number of serine residues in the TARP intracellular C-terminal domain via an indirect mechanism. Phosphorylation of the TARP C-terminal domain by CamKII inhibits its association with negatively charged phospholipids in the lipid bilayer, which in turn allows binding to PSD-95 and stabilization of receptors at the synapse (Sumioka et al., 2010). Dephosphorylation of these residues by the phosphatase PP1 (Tomita et al., 2005), downstream of NMDAR stimulation, favors association of the TARP intracellular domain with phospholipids, disrupting the TARP—PSD-95 interaction and consequently liberating the AMPAR from the confines of the PSD (Sumioka et al., 2010).

## Early Stages of Clathrin-Coated Pit Formation

### GluA2-AP2 Interaction

Following their dissociation from PSD scaffolds, it is thought that AMPARs diffuse from the synapse to EZs adjacent to the PSD (Lu et al., 2007). EZs have been defined by visualizing clusters of overexpressed fluorescently-tagged clathrin, and the structure of these sites with respect to the size or number of clathrin-coated pits (CCPs) present is unclear. One of the core elements of clathrin-mediated endocytosis (CME), and one of the first protein complexes to assemble at nascent CCPs, is the endocytic adaptor protein complex AP2, which functions to recruit and concentrate cargo at specific membrane domains. It clusters at PI(4,5)P_2_-rich regions of the plasma membrane, and binds cargo proteins, numerous endocytic accessory proteins and clathrin (Traub, 2009; Kelly and Owen, 2011). The μ2 subunit of AP2 binds GluA2 and GluA3 subunits directly (Figure 1C), and this interaction is required for hippocampal LTD but not constitutive AMPAR endocytosis (Lee et al., 2002; Kastning et al., 2007). The precise cell biological mechanism of AP2 binding to GluA2 has not been revealed, but by analogy with other well-studied cargo proteins, presumably it functions to recruit GluA2-containing AMPARs to endocytic sites (Traub, 2009; Kelly and Owen, 2011). Since it is involved in NMDAR-dependent endocytosis and not constitutive trafficking (Lee et al., 2002), the GluA2-AP2 interaction must be strengthened by NMDAR stimulation, although a mechanism has not been explored biochemically. Nevertheless, it has been suggested that AP2 binds the Ca^2+^ sensing protein hippocalcin, forming a Ca^2+^-dependent complex with AMPAR subunit GluA2 (Palmer et al., 2005). The AP2-hippocalcin interaction is required for LTD, suggesting that hippocalcin plays a role in recruiting AMPARs to endocytic sites in response to NMDAR-mediated Ca^2+^ signals.

### TARP-AP2 Interaction

As well as binding GluA2 directly, AP2 also associates with the AMPAR complex via TARPs (Matsuda et al., 2013; Figure 1C). As discussed above, while TARPs dissociate from the PSD scaffold in response to plasticity stimuli, they remain associated with the AMPAR complex, and continue to play an important role in AMPAR trafficking. Stargazin binds the μ2 subunit of AP2 via a C-terminal region that includes or overlaps with the region involved in regulating its association with phospholipids and hence with PSD-95 via stargazin phosphorylation (Sumioka et al., 2010; Matsuda et al., 2013). There are nine serine residues in this critical C-terminal region of Stargazin, and a specific subset of serines have been shown to modulate the binding of Stargazin to AP2 in response to NMDAR stimulation. Both cerebellar LTD and hippocampal LTD are disrupted by mutation of these serine residues (Tomita et al., 2005; Nomura et al., 2012). While it has been shown that PP1 causes an overall dephosphorylation of Stargazin and CamKII is involved in an overall increase in phosphorylation (Tomita et al., 2005), mutagenesis data suggest that AP2 binding increases when a cluster of three serines is dephosphorylated (experimentally, mutated to alanines). Other protein interactions with the Stargazin C-tail depend on different patterns of phospho-null or phospho-mimetic mutations in this region (Matsuda et al., 2013). The details of the upstream signaling pathways that converge on Stargazin to define these specific patterns of phosphorylation are unclear. Interestingly, one of the species of phospholipid that the Stargazin C-tail associates with in a protein phosphorylation-dependent manner is PI(4,5)P_2_, which is particularly concentrated at sites of endocytosis (Sumioka et al., 2010). Hence dephosphorylation of Stargazin may simultaneously promote association with AP2 and with PI(4,5)P_2_ in the plasma membrane. While disrupting binding to AP2 inhibited the NMDAR-dependent trafficking of recombinant Stargazin to early endosomes, it is unclear which stage of endocytosis leading up to this point is affected (Matsuda et al., 2013). Since binding to μ2 subunit of AP2 is typically associated with cargo recruitment to endocytic sites in the early stages of CCP formation, this is the most likely function for this interaction (Figure 1C). This leads to the question of why does μ2 subunit bind both GluA2 and Stargazin? Disrupting either of these interactions inhibits LTD, indicating that they are both important for activity-dependent AMPAR internalization (Lee et al., 2002; Matsuda et al., 2013). The number of TARPs that associate with an AMPAR complex has been suggested to vary (Greger et al., 2017). Perhaps the complement of TARPs associated with an AMPAR complex, and hence the number of μ2 binding sites, influences the speed or efficiency of AMPAR endocytosis? Moreover, while the vast majority of AMPARs contain GluA2 or GluA3 subunits, GluA1 homomers are thought to exist (Wenthold et al., 1996; Man, 2011). GluA1 does not bind μ2 (Kastning et al., 2007), hence the recruitment of these Ca^2+^-permeable AMPARs to CCPs might depend on their TARP-μ2 interactions, allowing for a subtly distinct mode of regulation compared to GluA2-containing AMPARs, which may be critical for specific kinds of plasticity that involve Ca^2+^-permeable AMPARs.

### PICK1-AP2 Interaction

While the μ2 subunit is critical for cargo recruitment, the appendage domain of the α subunit of AP2 (α-adaptin) binds several endocytic accessory proteins including amphiphysin, which contains a Bin-Amphiphysin-RVS (BAR) domain that senses or contributes to membrane curvature at the neck of the CCP and functions to recruit the large GTPase dynamin to the CCP neck for fission of the endocytic vesicle. (Praefcke et al., 2004; Daumke et al., 2014; Suetsugu et al., 2014). A recent addition to the BAR domain proteins identified as an α-appendage interactor is protein interacting with C-Kinase 1 (PICK1; Figure 1B; Fiuza et al., 2017), which has a well-established role in decreasing the surface and synaptic levels of GluA2-containing AMPARs (Terashima et al., 2004). The PICK1 PDZ domain binds the C-terminal tail of AMPAR subunit GluA2, and disrupting this interaction with competing peptides or by mutagenesis inhibits both constitutive and NMDAR-stimulated AMPAR internalization and LTD in hippocampal neurons (Daw et al., 2000; Osten et al., 2000; Iwakura et al., 2001), as well as cerebellar LTD. While a basal level of PICK1 appears to be bound to GluA2 to promote constitutive internalization, the interaction is enhanced directly by Ca^2+^ ions following NMDAR stimulation (Hanley and Henley, 2005). A direct effect of Ca^2+^ on GluA2-PICK1 binding, without the need for additional enzymatic steps, allows a rapid response to NMDAR stimulation. PICK1 contains at least two Ca^2+^ binding sites, one of which, a short stretch of acidic amino acids at the N-terminus of PICK1, is responsible for mediating the NMDAR-stimulated increase in GluA2 binding. Mutagenesis revealed that the Ca^2+^-binding property of PICK1 is necessary for NMDA-stimulated AMPAR internalization and LTD (Hanley and Henley, 2005; Citri et al., 2010).

PICK1 binds directly to AP2 with similar consensus motifs (FxDxF and DxF) to numerous other endocytic accessory proteins (Praefcke et al., 2004; Olesen et al., 2008; Fiuza et al., 2017). Mutating the critical aspartate residues to alanines in PICK1 disrupts AP2 binding and consequently inhibits both constitutive and NMDAR-dependent internalization of endogenous GluA2-containing AMPARs (Fiuza et al., 2017). While AP2-PICK1 binding is important for constitutive AMPAR internalization, NMDAR stimulation causes a marked increase in this interaction, which follows a slower time course compared to that of GluA2-PICK1, suggesting intermediate steps are involved in mediating the increase in binding, rather than a direct effect of Ca^2+^. Indeed, the NMDAR-dependent increase in AP2-PICK1 binding requires activation of the Ca^2+^-dependent phosphatase Calcineurin (Fiuza et al., 2017), which itself has a well-established role in NMDAR-dependent AMPAR internalization and LTD (Mulkey et al., 1994; Beattie et al., 2000). The substrate for Calcineurin in this mechanism is unknown. Furthermore, disrupting PICK1-AP2 binding blocks NMDAR-dependent recruitment of GluA2-containing AMPARs to clathrin clusters in neuronal dendrites, suggesting that PICK1 is involved in recruiting AMPARs to CCPs (Figure 1B). Mutagenesis of the PICK1 PDZ domain also blocks this trafficking event, indicating that AMPAR recruitment to endocytic sites also depends on PICK1 binding to GluA2 (Fiuza et al., 2017). However, α-adaptin and GluA2 binding to PICK1 are mutually exclusive, suggesting that the binding of both proteins simultaneously to PICK1 occurs only very transiently. Together, these observations indicate that PICK1 binds GluA2 immediately after NMDAR stimulation, followed by an increase in PICK1-AP2 binding, which consequently disrupts the interaction between PICK1 and GluA2 (Fiuza et al., 2017). While this suggests a mechanism for PICK1 in the recruitment of GluA2 to CCPs, the PICK1 interaction with α-adaptin is likely to be mechanistically distinct from the cargo recruitment function of the μ2 interactions. The α-appendage domains are found at the end of long flexible linker regions, which can reach out over a large area to bring in to the CCP accessory proteins required for inducing/sensing membrane curvature and recruiting dynamin (Praefcke et al., 2004). While PICK1 senses membrane curvature (Herlo et al., 2018) and binds dynamin (see following section), it also binds endocytic cargo. Hence, the PICK1—α-adaptin interaction may serve two functions; to enhance GluA2 clustering at CCPs because of the wide spatial sampling of the appendage domain, and to recruit a curvature-sensing regulator of dynamin.

### GluA1-Eps15 Interaction

Eps15 is a well-characterized endocytic adaptor protein that binds to and promotes the endocytosis of ubiquitinated cargo (Polo et al., 2002). Eps15 interacts with GluA1, and this interaction is enhanced by ubiquitination of the GluA1 C-terminal domain by the E3 ligase Nedd4 (Lin and Man, 2014). While Eps15 was shown to be required for glutamate-induced AMPAR endocytosis, a role for the GluA1-Eps15 interaction *per se* in this trafficking event has not been demonstrated. Furthermore, a number of reports suggest that AMPAR subunit ubiquitination is regulated by ligand (AMPA) stimulation, but not by NMDAR stimulation or other models of synaptic plasticity (Schwarz et al., 2010; Widagdo et al., 2015).

### GluA2-BRAG2 Interaction

The phospholipid composition of the plasma membrane is a critical determinant of AP2 clustering at nascent CCPs, since AP2 has high affinity for PI(4,5)P_2_ (Figures 1B,C). Hence a mechanism to locally increase PI(4,5)P_2_ concentration in the vicinity of AMPARs would promote AP2 binding to AMPAR subunits and associated proteins and hence facilitate endocytosis. Brefeldin-Resistant Arf-guanine nucleotide exchange factor 2 (BRAG2-GEF 2), a GEF for Arf6, binds directly to GluA2 at a site that includes Tyr 876 (Scholz et al., 2010; Figure 1B). Via this physical interaction, AMPAR stimulation increases BRAG2 GEF activity and consequently Arf6 activation in a mechanism that requires dephosphorylation of Y876. Arf6 is generally considered to function at the plasma membrane in recruiting lipid kinases to increase local concentration of PI(4,5)P_2_ for CCP formation (D’Souza-Schorey and Chavrier, 2006). Hence, PI(4,5)P_2_ levels might increase close to ligand-bound AMPARs, provided specific tyrosine phosphatases are activated to dephosphorylate Y876. However, such an effect on plasma membrane phospholipids in the context of AMPAR trafficking has not been reported. This process is required for mGluR-dependent AMPAR internalisation and LTD (Scholz et al., 2010). NMDAR-dependent LTD also requires BRAG2, but it is likely that a subtly different mechanism is at play between the two modes of LTD induction. Studies from other labs report tyrosine dephosphorylation of GluA2 as part of the mechanism for mGluR-dependent LTD, which is thought to require activation of the tyrosine phosphatase STEP downstream of mGluR stimulation (Moult et al., 2006; Zhang et al., 2008). In contrast, NMDAR-dependent LTD is thought to require phosphorylation of Y876 (Ahmadian et al., 2004; Hayashi and Huganir, 2004; and see later section).

## Later Stages of Clathrin-Coated Pit Formation; Bar Domains

A number of BAR domain proteins have been implicated in AMPAR endocytosis. Indeed, the first published evidence that LTD involves endocytosis was based on the use of a peptide corresponding to the amphiphysin SH3 domain to disrupt amphiphysin binding to dynamin, and hence inhibit dynamin recruitment to the CCP (Man et al., 2000). However, there appears to be no evidence to suggest that this interaction is regulated by NMDAR stimulation or other plasticity-inducing stimuli.

### PICK1-Dynamin Interaction

The PICK1 BAR domain is proposed to have a similar degree of curvature as amphiphysin, it contains two AP2 α-appendage binding sites (the same as amphiphysin), and it also binds dynamin (Figure 1D; Praefcke et al., 2004; He et al., 2011; Karlsen et al., 2015; Fiuza et al., 2017). The PICK1-dynamin interaction shows a similar dependence on NMDAR stimulation and calcineurin activity as PICK1-AP2, raising the possibility that PICK1 binds dynamin only as a functional consequence of binding AP2. Nevertheless, in a reduced system of purified components, PICK1 binds dynamin directly and enhances dynamin polymerization (Fiuza et al., 2017). The similar degree of curvature of the PICK1 BAR domain to amphiphysin is consistent with a role in recruiting dynamin to the highly curved neck of the CCP and regulating its function there, although this has not been shown experimentally. It is unknown whether the PICK1 BAR domain functions to induce or stabilize membrane curvature, or simply sense and associate with membranes of a particular curvature to recruit dynamin to the neck of the CCP. It is also unclear whether PICK1 and amphiphysin play distinct or redundant roles in dynamin recruitment at the AMPAR-containing CCP. While amphiphysin binds the proline-rich domain of dynamin (Ferguson and De Camilli, 2012), PICK1 binds the GTPase domain (Fiuza et al., 2017), suggesting distinct roles in regulating dynamin function. Note that PICK1 does not appear to play a role in AMPAR endocytosis associated with down-scaling homeostatic plasticity (Anggono et al., 2011).

### PACSIN-PICK1 Interaction

Another BAR domain protein shown to play a specific role in AMPAR endocytosis is PACSIN, also known as Syndapin. In contrast to the N-BAR domains of PICK1 or amphiphysin, PACSIN/Syndapin contains an F-BAR domain, which is elongated and has a preference for membranes with a larger radius of curvature (Qualmann et al., 2011). It is thought that F-BAR proteins are recruited to CCPs at an earlier stage of endocytosis compared to BAR or N-BAR proteins, in order to induce or stabilize the shallow curvature of the plasma membrane in the nascent CCP (Suetsugu et al., 2014). The precise temporal details of accessory protein recruitment to AMPAR-containing CCPs has not been specifically studied, however the recently-reported success at visualizing such events in neuronal dendrites with high temporal resolution suggests that progress in this direction will soon be made (Rosendale et al., 2017). PACSIN/Syndapin associates with AMPARs via an interaction with PICK1, and it has been suggested that phosphorylation of PACSIN/Syndapin at a cluster of three serines in the variable region between F-BAR and SH3 domains disrupts the interaction with PICK1 and reduces AMPAR internalization (Anggono et al., 2013). However, it has also been suggested that phosphorylation of the same three serines has more effect on recycling than on endocytosis of recombinant GluA2 (Widagdo et al., 2016). While knockdown of PACSIN/Syndapin expression reduces GluA2 endocytosis, indicating a critical role for the protein in this trafficking event, it is unclear whether any specific interaction with AMPARs or with AMPAR binding proteins is involved (Widagdo et al., 2016).

### Arc-Endophilin-CPG2-Actin Interactions

Endophilin is another BAR domain protein that functions in a similar manner as amphiphysin, associating with the neck of CCPs to regulate dynamin recruitment (Ferguson and De Camilli, 2012). A specific role for endophilin in AMPAR endocytosis has been demonstrated by the discovery of a direct interaction between endophilin and the immediate early gene Arc/Arg3.1 (Chowdhury et al., 2006). Although activity-dependent regulation of this interaction has not been reported, Arc/Arg3.1 gene expression is regulated by neuronal activity, and therefore the interaction with endophilin would be upregulated under conditions of increased gene expression. While the precise function of this interaction in endocytosis is unclear, Arc/Arg3.1 is required for both LTD and for down-scaling homeostatic plasticity (Rial Verde et al., 2006; Shepherd et al., 2006). Endophilin also associates with CPG2, another protein whose expression is regulated by neuronal activity (Loebrich et al., 2016). CPG2 in turn associates with the actin cytoskeleton, and both the endophilin-CPG2 and CPG2-actin interactions are required for homeostatic down-scaling (Loebrich et al., 2013, 2016). Phosphorylation of CPG2 by PKA enhances its interaction with the actin cytoskeleton, and disrupting this phosphorylation event inhibits AMPAR internalization, suggesting a phosphorylation-dependent regulation of AMPAR endocytosis via a protein complex comprising actin/CPG2/endophilin (Loebrich et al., 2013).

## The Actin Cytoskeleton

The role of the actin cytoskeleton in endocytosis is well-studied in the context of non-neuronal cells. Actin dynamics are proposed to generate forces that contribute to the changing geometry of the plasma membrane during CCP formation and to subsequent vesicle fission, and numerous proteins have been implicated in the regulation of this process (Kaksonen et al., 2006; Mooren et al., 2012). While it is likely that many of the same actin-binding protein players and consequent mechanisms are involved in regulating AMPAR endocytosis in neurons, there is little published evidence to support this directly. Nevertheless, it has been shown that the balance of actin polymerization and depolymerization is critical to AMPAR synaptic localization (Zhou et al., 2001).

### PICK1-Arp2/3 Interaction

While a number of actin-binding proteins associate directly or indirectly with AMPARs, they have not been reliably assigned a role in endocytosis *per se*, and there are very few publications reporting that such interactions are regulated by plasticity stimuli. One example is PICK1, which binds directly to the actin-nucleating Arp2/3 complex (Rocca et al., 2008). This interaction is transiently enhanced by NMDAR stimulation and is required for NMDA-induced AMPAR internalization and LTD (Nakamura et al., 2011). The signaling mechanism that mediates this NMDAR-dependent increase in binding involves the small GTPase Arf1, which associates with PICK1 in its GTP-bound state and blocks the interaction with Arp2/3 (Rocca et al., 2013). NMDAR stimulation switches Arf1 from a GTP- to GDP-bound state via the Arf GAP GIT1, and GDP-bound Arf1 dissociates from PICK1, promoting binding to Arp2/3 (Rocca et al., 2013). PICK1 inhibits Arp2/3-mediated actin polymerization, suggesting a requirement for inhibition of this activity at an unknown stage of AMPAR endocytosis (Rocca et al., 2008). The precise spatial and temporal details of this inhibition of actin polymerization are likely to be critical and warrant further study. Interestingly, a role for PICK1 inhibition of Arp2/3 activity and modulation by Arf1 has also been suggested recently in a specific form of endocytosis in non-neuronal cells (Sathe et al., 2018). In this study, the authors suggest that PICK1 functions to recruit inactive Arp2/3 to the sites of endocytosis, in preparation for a subsequent burst of actin polymerization triggered by the small GTPase Cdc42 and BAR domain protein IRSp53. However, a report from another group suggested that PICK1 does not bind to Arp2/3, but instead is involved in vesicle motility via an as yet undefined myosin motor protein (Madasu et al., 2015). A role for such an interaction in AMPAR endocytosis was not suggested.

## Protein-Protein Interactions That Modulate an Undefined Aspect of AMPAR Endocytosis

### GluA2-GRIP Interaction

The GRIP family of multi-PDZ domain scaffold proteins plays multiple roles in AMPAR trafficking, including long-range trafficking via association with microtubule motor proteins, endosomal sorting, and stabilization at the synaptic membrane (Osten et al., 2000; Setou et al., 2002; Steiner et al., 2005). GRIP binds GluA2 at the same site as PICK1, hence the two interactions are mutually exclusive and dissociation from GRIP1 is likely necessary prior to binding PICK1 and consequent endocytosis. The GluA2-GRIP interaction is modulated by phosphorylation of GluA2 at Serine 880, which lies within the PDZ ligand (Chung et al., 2000), and also by the nearby Tyr 876 (Hayashi and Huganir, 2004). Both phosphorylation events can be stimulated by NMDAR activation (Kim et al., 2001; Hayashi and Huganir, 2004). PICK1 binding is unaffected by S880 and Y876 phosphorylation, therefore these signaling events cause a switch of GluA2 binding from GRIP to PICK1 binding. S880 phosphorylation has been shown to be a critical component of both hippocampal and cerebellar LTD (Kim et al., 2001; Chung et al., 2003). While protein kinase C is required for phosphorylating S880 in cerebellar LTD, the kinase for hippocampal LTD is unknown (Xia et al., 2000; Kim et al., 2001).

### GluA2-Thorase and GluA2-NSF Interactions

A further mode of regulation of the GluA2-GRIP interaction is via the ATPase Thorase, whose activity is required for NMDAR-dependent GluA2 endocytosis and LTD (Zhang et al., 2011). Thorase binds both GluA2 and GRIP in an ATP-dependent manner, and its ATPase activity disrupts the GluA2-GRIP interaction to facilitate AMPAR endocytosis. Presumably the association of Thorase with the AMPAR-GRIP complex (or alternatively the enzymatic activity of Thorase) must itself be regulated by NMDAR activity, but such a mechanism has yet to be identified. Interestingly, a very similar, yet apparently independent mechanism regulates GluA2-PICK1 interactions. The ATPase NSF, well-characterized as a molecular chaperone for the SNARE complex, dissociates PICK1 from GluA2 in an ATP-dependent manner to limit AMPAR internalization (Hanley et al., 2002). Disrupting the GluA2-NSF interaction with competing peptides causes a rundown of AMPAR EPSCs that occludes subsequent expression of both hippocampal and cerebellar LTD (Luthi et al., 1999; Lee et al., 2002; Steinberg et al., 2004), suggesting that dissociation of this interaction is required for activity-dependent AMPAR internalization. In contrast to GluA2-Thorase, additional levels of modulation of the GluA2-NSF interaction have been identified. NSF binding to GluA2 is decreased in the presence of low-micromolar Ca^2+^, suggesting that NMDAR-mediated Ca^2+^ influx reduces the NSF-dependent dissociation of PICK1 from GluA2 (Hanley, 2007). In addition, the identity of the SNAP protein cofactor is a critical determinant of NSF activity on this complex; α-SNAP stimulates, whereas β-SNAP inhibits GluA2-PICK1 dissociation by NSF (Hanley et al., 2002).

## Concluding Remarks

I have reviewed what I believe to be the current state of knowledge about protein-protein interactions that are involved in AMPAR endocytosis from the plasma membrane and are regulated in response to stimuli that induce long-term synaptic plasticity. There exists a wealth of knowledge about the orchestration of protein-protein interactions in general endocytosis mechanisms, many of which are likely to be involved in AMPAR endocytosis. The complex signaling pathways that are activated in response to the induction of synaptic plasticity are also well characterized, hence the potential for regulating already-known endocytic protein-protein interactions as a consequence of plasticity stimuli is significant and worthy of future investigation. Furthermore, it is emerging that the dysregulation of AMPAR endocytosis is a critical component of synaptic weakening associated with pathologies such as Alzheimer’s, and therefore dynamic protein-protein interactions might become targets for therapeutic intervention.

## Author Contributions

The author confirms being the sole contributor of this work and has approved it for publication.

## Conflict of Interest Statement

The author declares that the research was conducted in the absence of any commercial or financial relationships that could be construed as a potential conflict of interest.
